# A Recombinant Vesicular Stomatitis Virus-Based Lassa Fever Vaccine Protects Guinea Pigs and Macaques against Challenge with Geographically and Genetically Distinct Lassa Viruses

**DOI:** 10.1371/journal.pntd.0003736

**Published:** 2015-04-17

**Authors:** David Safronetz, Chad Mire, Kyle Rosenke, Friederike Feldmann, Elaine Haddock, Thomas Geisbert, Heinz Feldmann

**Affiliations:** 1 Laboratory of Virology, Division of Intramural Research, Rocky Mountain Laboratories, National Institute of Allergy and Infectious Diseases, National Institutes of Health, Hamilton, Montana, United States of America; 2 Galveston National Laboratory and Department of Microbiology and Immunology, University of Texas Medical Branch, Galveston, Texas, United States of America; 3 Rocky Mountain Veterinary Branch, Division of Intramural Research, Rocky Mountain Laboratories, National Institute of Allergy and Infectious Diseases, National Institutes of Health, Hamilton, Montana, United States of America; Tulane School of Public Health and Tropical Medicine, UNITED STATES

## Abstract

**Background:**

Lassa virus (LASV) is endemic in several West African countries and is the etiological agent of Lassa fever. Despite the high annual incidence and significant morbidity and mortality rates, currently there are no approved vaccines to prevent infection or disease in humans. Genetically, LASV demonstrates a high degree of diversity that correlates with geographic distribution. The genetic heterogeneity observed between geographically distinct viruses raises concerns over the potential efficacy of a “universal” LASV vaccine. To date, several experimental LASV vaccines have been developed; however, few have been evaluated against challenge with various genetically unique Lassa virus isolates in relevant animal models.

**Methodologies/principle findings:**

Here we demonstrate that a single, prophylactic immunization with a recombinant vesicular stomatitis virus (VSV) expressing the glycoproteins of LASV strain Josiah from Sierra Leone protects strain 13 guinea pigs from infection / disease following challenge with LASV isolates originating from Liberia, Mali and Nigeria. Similarly, the VSV-based LASV vaccine yields complete protection against a lethal challenge with the Liberian LASV isolate in the gold-standard macaque model of Lassa fever.

**Conclusions/significance:**

Our results demonstrate the VSV-based LASV vaccine is capable of preventing morbidity and mortality associated with non-homologous LASV challenge in two animal models of Lassa fever. Additionally, this work highlights the need for the further development of disease models for geographical distinct LASV strains, particularly those from Nigeria, in order to comprehensively evaluate potential vaccines and therapies against this prominent agent of viral hemorrhagic fever.

## Author Summary

Lassa fever (LF) is an acute viral infection which is often associated with hemorrhagic manifestations and multi-organ failure in humans. The etiological agent responsible for LF is Lassa virus (LASV), a rodent-borne Arenavirus which is endemic in several West African countries. Up to 500,000 cases of LF are diagnosed annually, primarily in Nigeria, Liberia, Guinea and Sierra Leone. The high incidence rate combined with the significant morbidity and mortality associated with LASV infection highlights the need for an effective prophylactic vaccine for LF. Importantly, an ideal LASV vaccine should provide protection against genetically and geographical divergent viral strains. Previously a recombinant vesicular stomatitis virus (VSV)-based LF vaccine using the glycoproteins of LASV strain Josiah as the immunogen, was shown to completely protect non-human primates against a homologous (LASV strain Josiah) challenge. Here, we have expanded the original studies and tested the VSV-LASV vaccine against challenge with LASV isolates from Mali, Liberia and Nigeria in the strain 13 guinea pig and cynomolgus macaque disease models. Our results suggest that the VSV-based LF vaccine affords complete protection against geographically and genetically distinct viral isolates.

## Introduction

Lassa virus (LASV, family Arenaviridae, genus *Arenavirus*) is one of the most prominent etiological agents of viral hemorrhagic fever with an estimated 300,000–500,000 infections diagnosed annually in West Africa [1, 2]. Humans are most commonly infected with LASV through contact with infected rodents (*Mastomys natalensis*) or virus-contaminated rodent excreta/secreta, although person-to-person transmission can occur, especially in hospital settings. Clinically, LASV infections range from apparently asymptomatic or mild to severe hemorrhagic fever characterized by multi-organ failure, a condition referred to as Lassa fever (LF) [1]. Overall, mortality rates associated with LASV are approximately 2%; however this rate increases to 15% for hospitalized cases and can exceed 50% in outbreak scenarios [2, 3]. Despite the high incidence and associated morbidity and mortality, there is no approved vaccine to prevent LASV infection or disease in humans. Currently, treatment of LF cases relies on supportive care and, where available, the early administration of ribavirin [4].

Geographically, LASV is restricted to West Africa and has two endemic regions: Sierra Leone, Guinea and Liberia and Nigeria, where LASV infections are annually documented [5–9]. Sequence analysis of LASV isolates from these regions show a remarkably high level of genetic diversity, with at least four lineages of LASV described that correlate with geographical regions [10]. LASV clades I, II and III are localized in Nigeria and appear to be ancestral to clade IV viruses which are found in and around Sierra Leona, Liberia and Guinea. The genetic heterogeneity within LASV raises questions about the efficacy of a potential universal LASV vaccine which could elicit a protective immune response capable of preventing infection from all strains of LASV.

To date, several experimental platforms have been evaluated as potential LF vaccines with most candidates utilizing the LASV glycoproteins and/or nucleocapsid protein as immunogens. These include inactivated LASV particles, virus-like particles, peptides, recombinant DNA, RNA replicon vectors, recombinant vaccinia viruses, a reassortant Mopeia virus/LASV (ML29), and a recombinant yellow fever 17D vaccine expressing LASV glycoproteins [11–21]. While the majority of these platforms demonstrated a robust immune response in laboratory animals, many have failed to fully protect against lethal disease in relevant disease models and only a few have been assessed against a non-homologous LASV challenge [22].

Recently, a recombinant, replication-competent, vesicular stomatitis virus (VSV)-based LF vaccine expressing the glycoproteins of LASV strain Josiah (VSVΔG/LASVGPC) was shown to protect non-human primates against a lethal challenge with the homologous LASV Josiah [23]. The goal of these studies was to further evaluate the VSV platform as a potential LF vaccine. Specifically, we sought to determine if, like other LF vaccine platforms, a VSV-based vaccine which expresses the LASV nucleocapsid antigen would provide protection against lethal LASV challenge [19, 21, 24]. Furthermore, we wanted to assess the ability of the VSV-based LF vaccines to protect inbred guinea pigs and macaques against challenge with genetically-unique (non-homologous) LASV isolates originating from Liberia, Mali and Nigeria. Our results demonstrate the VSVΔG/LASVGPC LF vaccine is superior to a VSV-based LF vaccine which utilizes the nucleocapsid protein as an immunogen and the GPC-based vaccine is capable of preventing morbidity and mortality associated with infection by geographically and genetically distinct LASVs in two animal models of disease

## Methods

### Ethics statement

All experiments were approved by the Institutional Animal Care and Use Committee of the Rocky Mountain Laboratories (RML 2010–61, 2011–30). Animal work was conducted adhering to the institution’s guidelines for animal use, and followed the guidelines and basic principles in the United States Public Health Service Policy on Humane Care and Use of Laboratory Animals, and the Guide for the Care and Use of Laboratory Animals by certified staff in an Association for Assessment and Accreditation of Laboratory Animal Care, International (AAALAC) accredited facility.

### Biosafety

All work with infectious LASV and potentially infectious materials derived from animals was conducted in a Biosafety Level 4 (BSL 4) laboratory in the Integrated Research Facility of the Rocky Mountain Laboratories (RML), National Institute of Allergy and Infectious Diseases (NIAID), National Institutes of Health (NIH). Sample inactivation and removal was performed according to standard operating protocols approved by the local Institutional Biosafety Committee.

### Viruses

Low passage (passage four or less) LASV isolates Josiah (Sierra Leona, clade IV) [25], Z-132 (Liberia, clade IV) [26], Soromba-R (Mali, clade IV) [27] and Pinneo (Nigeria, clade I) [28] were cultured in Vero cells and titered using standard methodologies.

### VSV vaccines

The recombinant VSV vectors expressing the glycoproteins of LASV Josiah (VSVΔG/LASVGPC) or the glycoproteins of Andes virus (VSVΔG/ANDVGPC) were propagated and titrated as previously described [23, 29]. The control vaccine (VSVΔG/ANDVGPC) was selected for these studies because both constructs have similar growth kinetics *in vitro*. Using RNA isolated from infected Vero cells, the construction of cDNA containing the nucleocapsid protein (NP) from LASV strain Josiah was accomplished using reverse transcription with a gene specific primer recognizing the 3′ end of the NP gene in the LASV genome. This cDNA was subsequently used to amplify the NP gene by PCR with primers containing *Pac*I and *Asc*I at the 5′ and 3′ ends respectively. Creation of the full-length recombinant VSV genome with LASV NP was accomplished by cloning the sequence into the plasmid pVSV 4.1 (kindly provided by Dr. John Connor), which encodes a VSV anti-genome that has a multiple cloning site between the M protein and G protein coding sequences. The plasmid and PCR amplified fragment were digested with *Pac*I and *Asc*I; the vector and fragment were gel purified and ligated to create pVSV-LASV-NP. The recombinant virus (VSVG/LASVNP) was recovered using reverse genetics [30] with some modifications as described previously [31].

### Homologous challenge guinea pig studies

Adult inbred, strain 13 guinea pigs (*Cavia porcellus*, aged 2 to 4 months) were obtained from an in-house breeding colony and randomly distributed into experimental groups. To compare the protective efficacy of prophylactic immunization with vectors expressing the LASV glycoproteins versus those expressing the nucleocapsid protein, two groups of nine animals were immunized with 1 x10^6^ plaque forming units (pfu) of VSVΔG/LASVGPC or VSVG/LASVNP via intra-peritoneal (i.p.) injection. A control group of four guinea pigs was similarly immunized with an irrelevant VSV vector expressing the glycoproteins of Andes virus. At 28 days post-immunization, animals were challenged with 1 x10^4^ 50% tissue culture infective doses (TCID_50_) of LASV strain Josiah and monitored daily for signs of disease (activity, posture, recumbence, respiration, weight loss). At the time when control animals were euthanized due to disease severity, three animals immunized with VSVΔG/LASVGPC and VSVG/LASVNP were exsanguinated and tissue samples (lung, liver, spleen and blood) collected to determine the extent of viral replication. Animals that did not develop lethal disease were monitored for 45 days post-inoculation and exsanguinated.

### Non-homologous guinea pig challenge studies

Two groups of nine guinea pigs were immunized with 1 x10^6^ pfu VSVΔG/LASVGPC as outlined above and challenged 28 days later with 1 x10^4^ TCID_50_ of LASV isolates Z-132 or Soromba-R. Control animals (n = 5 for Z-132 challenge and n = 9 for Soromba-R challenge) were immunized with VSVΔG/ANDVGPC as outlined above and challenged with the appropriate LASV strain according to the same schedule. At the time when control animals were euthanized due to disease severity, three animals immunized with VSVΔG/LASVGPC and challenged with LASV Z-132 were euthanized and samples collected for viral titration. However, because LASV Soromba-R is not 100% lethal in the guinea pig model, all animals were monitored for signs of disease and survival. Animals that did not develop lethal disease were monitored for 45 days post-inoculation and exsanguinated with sera collected to assess seroconversion. In a follow-up study, groups of 8 or 7 guinea pigs were immunized with VSVΔG/LASVGPC or VSVΔG/ANDVGPC and challenged with LASV Pinneo, genetically one of the most divergent LASV isolates described, as outlined above. To date there is little information available on infection of inbred guinea pigs with LASV Pinneo, therefore, similar to the Soromba-R challenge study, all animals were monitored for disease progression and survival.

### LASV Z-132 (non-homologous) non-human primate challenge study

Four female cynomolgus macaques (*Macaca fascicularis* aged 4 to 4.5 years old, weights between 3 and 4.5 kg) were housed in individual metal cages in a dedicated room in the RML BSL 4 facility according to approved standard operating procedures for high containment. The room’s environment was strictly controlled by automated systems and was maintained at 24°C +/- 2°C with 50% humidity and 15 air changes per hour. Animals were fed commercially produced monkey chow (25% protein, Harlan) twice daily and had unlimited access to drinking water via an automated watering system. Environmental enrichment included toys, mirrors, treats and fruit, and cages were arranged such that animals maintained visual and auditory contact with one another. All experimental manipulations and clinical exams were performed on sedated animals (10–15 mg/kg ketamine delivered by intramuscular injection). Macaques were immunized intramuscularly with a single dose of 6 x10^7^ pfu of either VSVΔG/LASVGPC (n = 3) or VSVΔG/ANDVGPC (n = 1). At 28 days post-immunization, animals were challenged by intramuscular injection with a previously determined lethal dose (1 x10^4^ TCID_50_) of LASV strain Z-132 [32]. Following immunization and continuing for the duration of the study, animals were monitored twice daily and scored for adverse reactions to immunization or clinical signs of disease using an approved scoring sheet (fever, posture, respiration, feces/urine, food intake, recumbence, attitude, and skin turgor). Clinical exams were conducted on anesthetized animals on days -28, -18, 0, 1, 4, 7, 10, 14 and continued weekly until day 28 after which time survivors were monitored daily, but not sampled until the end of the study (day 42 post-inoculation). During exams the health status of each animal was assessed and blood samples were collected for analysis of blood chemistry, coagulation parameters, differential cell count and virus detection. Terminally ill animals were euthanized in accordance with the AVMA guidelines and the recommendations of the Weatherall report while under anesthesia with a pentobarbital-based solution and necropsies performed with collection of clinical specimens from lung, heart, liver and spleen for histological analysis and virus titrations.

### Hematology, serum biochemistries and coagulation parameters

Hematological analysis was measured in EDTA blood with the HemaVet 950FS+ hematology analyzer (Drew Scientific, Waterbury, CT). Serum biochemistries were monitored using a Piccolo Blood Analyzer (Abaxis, Sunnyvale, CA). Fibrinogen concentration and activated partial thromboplastin time (aPTT) were measured in citrate plasma samples on a STart4 instrument using the PTT and Fibri-Prest automate reagents (all from Diagnostica Stago, Parsippany, NJ), respectively. D-dimer concentrations were determined in plasma samples using the Asserachrom D-Di enzyme-linked immunosorbent assay (Diagnostica Stago). All assays were run on serial samples collected from infected NHPs, but not guinea pigs.

### LASV detection

Total RNA was extracted from blood samples using QIAamp viral RNA extraction kits (Qiagen, Valencia, CA) and tested for the presence of LASV RNA using a nested pan-Old World arenavirus RT-PCR assay targeting the viral polymerase gene, as previously described [33]. Infectious virus titers from tissue homogenates (10% wt/vol) and RT-PCR positive blood samples were determined in triplicate with 10-fold serial dilutions on Vero 76 cells using standard TCID_50_ methodologies and the Reed-Muench formula.

### Serology

Convalescent serum samples collected from surviving guinea pigs and NHPs at 45 and 42 days post-inoculation, respectively, were tested for the presence of IgG antibodies reactive to the LASV nucleocapsid protein using an in-house enzyme-linked immunosorbent assay (ELISA) based on a bacterially expressed recombinant antigen using standard methodologies. Briefly, 96-well polyvinyl plates were coated with 2ug of recombinant antigen suspended in 100ul of PBS. The following day plates were washed (PBS with 0.05% Tween-20) three times and 100ul of samples (4-fold dilutions, 1:100 to 1:6400 diluted in PBS with 5% skim milk powder) were added to duplicate wells and incubated at room temperature for 1 hour. Plates were washed as above after which the appropriate secondary antibody (HRP-labelled goat anti-nonhuman primate or goat-anti guinea pig, 1:1000 dilution both from KPL, Gaithersburg, MD) was added and incubated for 1 hour at room temperature. Plates were washed again and substrate added (ABTS peroxidase substrate, KPL) according to the manufacturer’s specifications for 15 minutes in the dark. Optical densities were read at 405nm and samples were considered positive if they had an OD_405_>3 standard deviations above of the average value of a panel of 12 known negative samples.

### Statistical analysis

Survival rates were compared using Fisher’s exact test with GraphPad QuickCalcs software.

## Results

### VSVΔG/LASVGPC provides greater protection than VSVG/LASVNP against homologous challenge in guinea pigs

**Fig 1:**
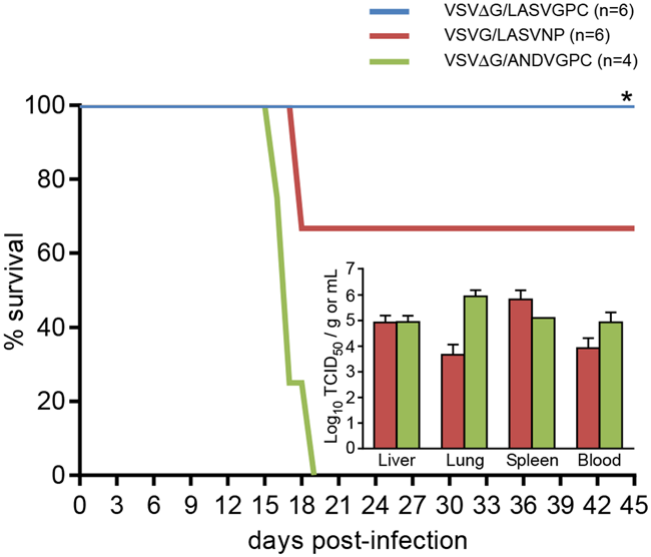
VSVΔG/LASVGPC affords better protection than VSVG/LASVNP against a lethal homologous challenge in strain 13 guinea pigs. Adult, inbred strain 13 guinea pigs were immunized with 1 x10^6^ pfu of VSVΔG/LASVGPC (n = 9), VSVG/LASVNP (n = 9) or VSVΔG/ANDVGPC (n = 4) and challenged 28 days later with a lethal dose of Lassa virus strain Josiah. Animals were monitored for disease progression for 45 days. At the time when control (VSVΔG/ANDVGPC immunized) animals demonstrated signs of advanced disease requiring euthanasia, three animals per group were euthanized and samples collected for virological analysis (inset). * p = 0.0048

In the first experiment we sought to determine if immunization with a vaccine construct expressing the LASV nucleocapsid protein would provide similar levels of protection as a construct expressing the glycoproteins. Following challenge with LASV Josiah, mock-immunized guinea pigs began showing signs of disease including lethargy and weight loss around day 12, which progressed to terminal disease in all animals between days 16 and 18. Similar to the original description of the VSV LASV vaccine [23], animals immunized with VSVΔG/LASVGPC were completely protected against challenge with LASV Josiah and did not demonstrate any signs of disease throughout the course of the experiment (p = 0.0048). In contrast, all animals immunized with VSVG/LASVNP demonstrated signs of infection, including lethargy and weight loss averaging between 10 and 20% of the initial body weight. Disease progression in these animals mirrored that of the control group and ultimately two of six animals in the vaccinated group succumbed to infection, resulting in a survival rate of 66.6% (p = 0.0714, Fig 1). Analysis of viral loads in tissue samples supported the survival data in that infectious LASV could not be isolated from any sample collected from animals immunized with VSVΔG/LASVGPC, whereas tissue titers from animals immunized with VSVG/LASVNP were similar to those of the controls (Fig 1, inset). Although the VSVG/LASVNP construct afforded some protection against lethality in the guinea pig model, all animals experienced significant morbidity. Therefore, the subsequent non-homologous challenge experiments were conducted only with VSVΔG/LASVGPC.

### VSVΔG/LASVGPC protects guinea pigs against clade IV LASV challenge

**Fig 2:**
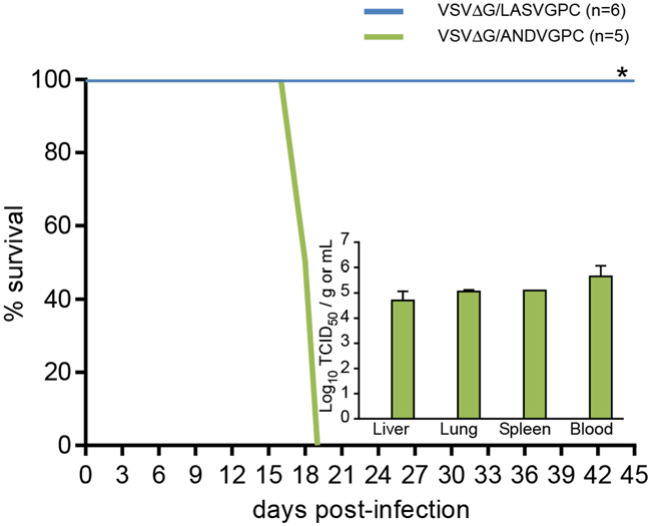
VSVΔG/LASVGPC protects strain 13 guinea pigs from lethal challenge with a Liberian Lassa virus isolate. Adult, inbred strain 13 guinea pigs were immunized with 1 x10^6^ pfu of VSVΔG/LASVGPC (n = 9) or VSVΔG/ANDVGPC (n = 5) and challenged 28 days later with a lethal dose of Lassa virus strain Z-132. Animals were monitored for disease progression for 45 days. At the time when control (VSVΔG/ANDVGPC immunized) animals demonstrated signs of advanced disease requiring euthanasia, three animals per group were euthanized and samples collected for virological analysis (inset). * p = 0.0022.

**Fig 3:**
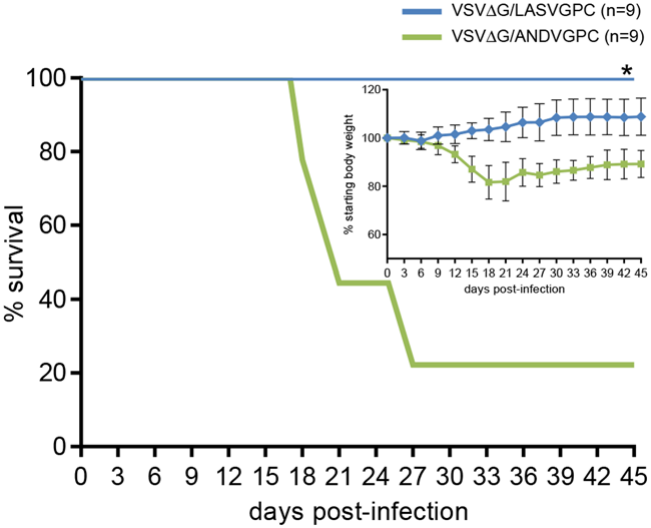
VSVΔG/LASVGPC protects strain 13 guinea pigs from infection / disease following challenge with a Malian Lassa virus isolate. Adult, inbred strain 13 guinea pigs were immunized with 1 x10^6^ pfu of VSVΔG/LASVGPC (n = 9) or VSVΔG/ANDVGPC (n = 9) and challenged 28 days later with Lassa virus strain Soromba-R. Animals were monitored for disease progression including weight loss for up to 45 days. * p = 0.001.

Similar to the results of the homologous challenge experiment outlined above, VSVΔG/LASVGPC completely protected guinea pigs against challenge with non-homologous LASV isolates originating from geographically distinct regions of West Africa (Fig 2). As previously observed, LASV Z-132 was 100% lethal within 18 days post-inoculation in control (VSVΔG/ANDVGPC)-immunized strain 13 guinea pigs [32]. In contrast, animals immunized with VSVΔG/LASVGPC were 100% protected against the Liberian LASV with no signs of disease observed throughout the course of the experiment (p = 0.0022). Furthermore, infectious virus could not be isolated from tissue samples collected from representative animals euthanized and sampled during the terminal phase of disease in the experimental group (Fig 2, inset). Similarly, animals immunized with VSVΔG/LASVGPC completely resisted challenge with the Malian LASV isolate and although (as previously described) Soromba-R is not 100% lethal in naïve animals [32], there was a statistically significant increase in the survival rate associated with VSVΔG/LASVGPC immunization (p = 0.001) (Fig 3)

### VSVΔG/LASVGPC protects guinea pigs against infection with the divergent clade I LASV Pinneo

**Fig 4:**
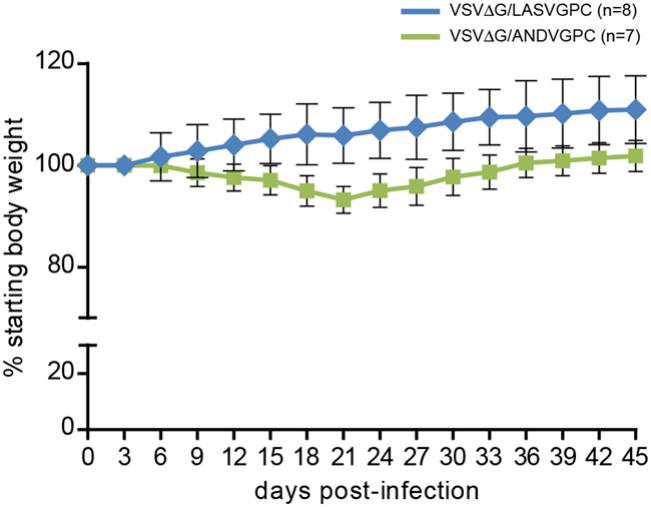
VSVΔG/LASVGPC protects strain 13 guinea pigs from infection following challenge with a highly divergent Nigerian Lassa virus isolate. Adult, inbred strain 13 guinea pigs were immunized with 1 x10^6^ pfu of VSVΔG/LASVGPC (n = 8) or VSVΔG/ANDVGPC (n = 7) and challenged 28 days later with Lassa virus strain Pinneo. Animals were monitored for disease progression including weight loss for up to 45 days.

In a final experiment, immunized guinea pigs were challenged with LASV Pinneo, a Nigerian LASV isolate which is among the most genetically divergent when compared to LASV Josiah [10]. Following infection, control (VSVΔG/ANDVGPC)-immunized animals demonstrated mild to moderate signs of disease, including lethargy and weight loss of between 5 and 10% of the starting body weight beginning around day 10 post-challenge. These signs of disease persisted until approximately day 22 at which point all animals recovered (Fig 4). In contrast, none of the animals immunized with VSVΔG/LV-GPC demonstrated any signs of disease throughout the course of the study. Supporting this, serological analysis conducted on convalescent serum samples revealed the presence of anti-LASV nucleocapsid IgG antibodies in the control group with titers between 1600 (n = 1) and ≥6400 (n = 6), while serological titers for VSVΔG/LASVGPC immunized animals were considerably lower ranging from equivocal (100, n = 5) to 400 (n = 3) (Table 1).

**Table 1:** End-point ELISA titers for the determination of seroconversion in immunized and control strain 13 guinea pigs post-Lassa virus challenge.

Challenge virus	Vaccine	No. Survivors (%)	End point ELISA (anti-NP) titters
LASV Josiah	VSVΔG/LASVGPC	6 (100%)	<100 (n = 6)
LASV Josiah	VSVΔG/ANDVGPC	0 (0%)	n.d.
LASV Z-132	VSVΔG/LASVGPC	6 (100%)	100 (n = 2), 400 (n = 3), 1600 (n = 1)
LASV Z-132	VSVΔG/ANDVGPC	0 (0%)	n.d.
LASV Soromba-R	VSVΔG/LASVGPC	9 (100%)	100 (n = 5), 400 (n = 4)
LASV Soromba-R	VSVΔG/ANDVGPC	2 (22.2%)	1600 (n = 2)
LASV Pinneo	VSVΔG/LASVGPC	8 (100%)	100 (n = 5), 400 (n = 3)
LASV Pinneo	VSVΔG/ANDVGPC	7 (100%)	1600 (n = 1), ≥6400 (n = 6)

### VSVΔG/LASVGPC protects NHPs against lethal Liberian LASV challenge

**Fig 5:**
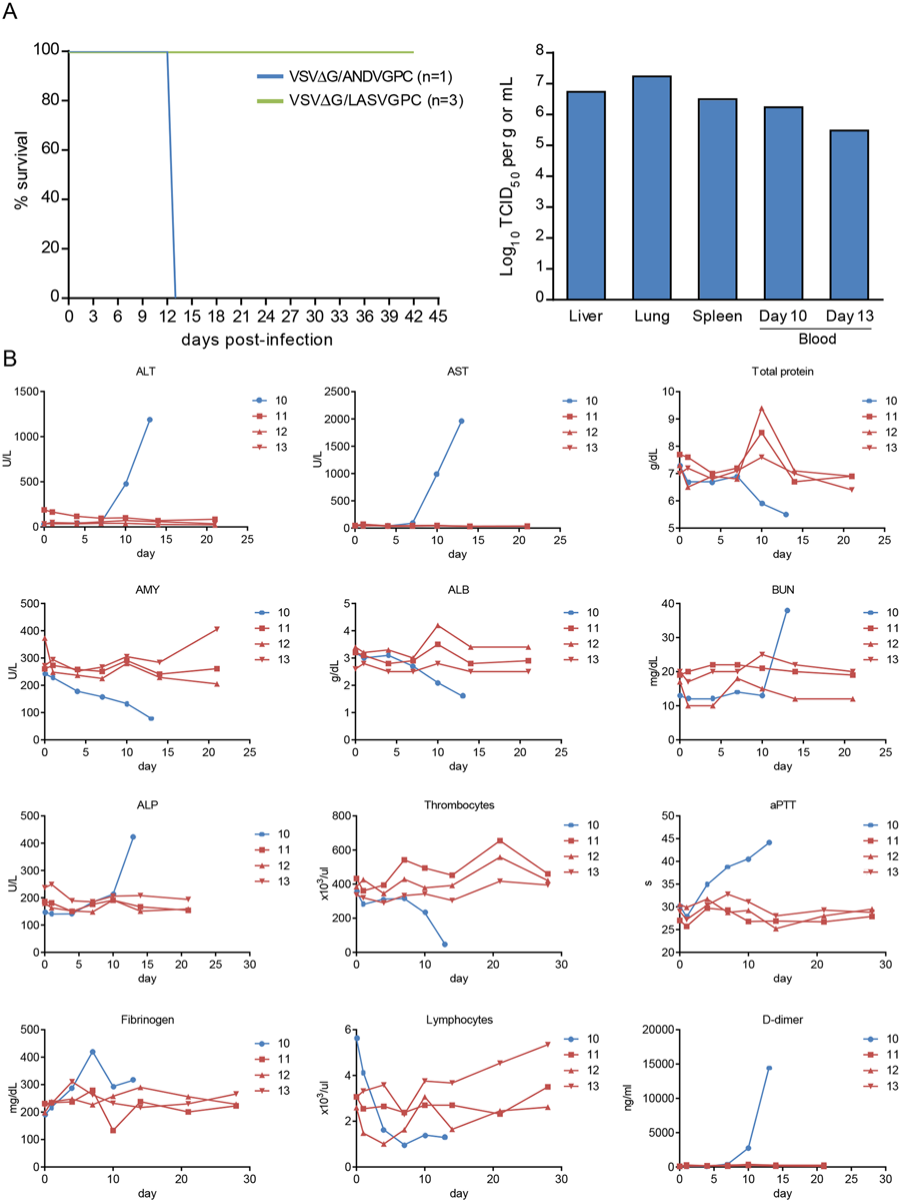
VSVΔG/LASVGPC protects non-human primates from a lethal challenge with a Liberian Lassa virus isolate. Cynomolgus macaques were immunized with 1 x10^7^ pfu of VSVΔG/LASVGPC (n = 3) or VSVΔG/ANDVGPC (n = 1) and challenged 28 days later with a previously determined lethal dose of Lassa virus strain Z-132. **(A)** Survival curve and infectious titers of Lassa virus in select tissues from the control animal collected at the time of euthanasia. **(B)** Selected hematological and biochemical parameters monitored from vaccinated (red lines) and control (blue line) macaques.

Based on the similarities to human disease, the apex animal model for viral hemorrhagic fevers, including LF, is widely considered to be NHPs. To further evaluate the VSV LF vaccine, three NHPs were immunized with VSVΔG/LASVGPC, while a single control NHP was immunized with an irrelevant VSV construct. Following immunization, all four animals appeared normal, with no clinically significant changes observed in any of the monitored parameters. At 28 days post-immunization, animals were challenged with a previously determined lethal dose of a non-homologous Liberian LASV (strain Z-132) [32]. Between days 5 and 7 post-infection the control animal began to demonstrate general signs of illness (reduced food-take, hunched posture, dull appearance, ruffled fur and reluctance to move) which progressed until day 13 at which point the animal was humanely euthanized (Fig 5A). At necropsy the animal presented with gross pathological abnormalities which were indicative of systemic disease; these included splenomegaly, hepatomegaly, pericardial effusion and slight pulmonary discoloration. Viral titrations (TCID_50_) confirmed the presence of LASV in selected organs (lung, liver and spleen) from the control animal, with organ titers ranging from 5.5 to 7.25 log_10_ TCID_50_’s per gram of tissue (Fig 5A). In contrast, the three NHPs immunized with VSVΔG/LASVGPC survived challenge with the Liberian LASV with no apparent signs of infection observed throughout the course of the study. The survival rate was statistically significant (100% versus 0%, p = 0.0286 by Fisher’s exact test) when one compares the three VSVΔG/LASVGPC-immunized animals to the single control animal utilized in this study combined with three previous control animals infected with LASV Z-132 (same route and dose) which succumbed to infection between days 10 and 13 post-challenge [32].

Analysis of hematology, serum biochemistries and selected coagulation parameters were performed on blood samples collected during exams. Considerable increases were noted in ALT, AST and ALP in the control animal as well as clinically significant decreases in albumin and total protein (Fig 5B). In addition, changes were observed in amylase (decrease) and BUN (drastic perimortem increase). Similar to previous reports with Z-132 challenge in NHPs, lymphopenia and thrombocytopenia were also documented in the control animal as well as increases in aPTT, fibrinogen, and D-dimer concentrations [32] (Fig 5B). Values from the three VSVΔG/LASVGPC-immunized animals remained relatively static throughout the course of the study, with only a minimal increase in total WBC counts and a transient spike in total protein observed on day 10 post-challenge. Viremia was noted only in the control animal, with infectious virus titers ranging between 5.5 and 6.25 log_10_ TCID_50_’s/mL of blood on days 10 and 13 post-infection. At no point during the study was LASV detected in blood samples from the three VSVΔG/LASVGPC-immunized animals, even when analyzed using a sensitive, nested RT-PCR approach.

## Discussion

There is an urgent need for the development of prophylactic countermeasures to prevent LASV infections and LF. According to a recent World Bank census, the combined population of Sierra Leone, Guinea, Liberia and Nigeria, the historic endemic region of LF, exceeds 190 million people [34]. However, the identification of LF cases and/or LASV-infected rodents in an expanded region, including Burkina Faso, Cote d’Ivoire, Mali, Benin and Ghana, suggest a larger region of LASV endemicity, and therefore a larger population at risk of contracting LASV infection [9, 27, 35]. In addition, over the last several decades imported cases of LF have been documented in the United States, Canada, the United Kingdom, Germany, the Netherlands, Israel and Japan. The appearance of these cases, combined with the classification as a Category A pathogen and concerns of use as a biological weapon, make LASV a global public health problem [36]. Despite the high incidence of infection, the large population at risk, and the concern of an intentional release, currently no approved LASV vaccine exists.

To date several experimental platforms have been evaluated as potential LF vaccines, however most of these have only been evaluated against homologous challenge. A notable exception is ML29 which was shown to protect guinea pigs against an otherwise lethal challenge with a non-homologous LASV isolate from Nigeria (LASV-803213) [37]. While these results are promising, the make-up of ML29, a reassortant arenavirus containing the small genomic segment (encoding the glycoproteins and nucleocapsid protein) of LASV Josiah and the large genomic segment (encoding the polymerase and matrix proteins) of Mopeia virus, qualifies it as a CDC risk group 3 pathogen in North America which may inhibit clinical trials in humans. Noteworthy, a VSV-based Ebola vaccine is already in clinical trials which may pave the way for future trials with the LF and other VSV-based vaccines. Importantly, although it is now evident that Ebola and LASV have overlapping regions of endemicity, it was recently demonstrated that sequential immunization with the VSV-based LF and Ebola vaccines does not alter their overall protective efficacy [38]. These findings suggest that the use of both of these leading vaccine candidates in a single population would not be a disadvantage.

Interestingly, unlike in other vaccine platforms, the LASV nucleocapsid antigen was not as potent of an immunogen in the VSV-platform as was the glycoproteins. Previous work with alphavirus-replicon particles and recombinant vaccinia viruses expressing the LASV nucleocapsid protein demonstrated increased survival over the rates reported here with VSVG/LASVNP; however it should be noted that at least with the vaccinia system, protection was not always complete [19, 21, 24]. While this phenomenon requires further study, the differences in the protective immune responses associated with immunization of these nucleocapsid-expressing vaccines is most likely reflective of the different arms of the immune system which are activated by the various platforms. Studies are currently underway to evaluate humoral versus cellular protective immune responses associated with the VSV LF vaccine.

Previously the VSV-based LF vaccine which expresses the glycoproteins of LASV strain Josiah has been shown to completely protect NHPs against lethal homologous challenge. Here we demonstrate that prophylactic immunization with the recombinant VSV-based LF vaccine protects inbred guinea pigs from infection / disease associated with several LASV isolates; in addition it protects NHPs from a lethal challenge with a virulent LASV strain from Liberia. The results of this study suggest a single LASV vaccine can be effective in preventing LF in Sierra Leone, Liberia and Mali as well as other countries, including Guinea, Burkina Faso and Cote d’Ivoire, where genetically similar clade IV LASVs are circulating. Importantly, immunization with the Josiah-based VSV vaccine appears to sterilely protect animals against lethal challenge with other clade IV viruses as demonstrated by an absence of clinical signs and no detectable infectious virus in tissue or blood samples in these animals. Similar results were observed in the Pinneo infection model with no signs of disease observed in any of the immunized guinea pigs and a lack of seroconversion in 5 of 8 animals. These promising preliminary results suggest further evaluation of the protective efficacy of the Josiah-based LF vaccine against Nigerian LASV strains should be conducted. However, to achieve this goal the development and characterization of appropriate disease models for Nigerian LASV strains, particularly clade II and III viruses which are frequently associated with human disease, must first be accomplished.
